# COVID-19 and the selection problem in national cause-of-death statistics

**DOI:** 10.1007/s40656-021-00420-8

**Published:** 2021-05-25

**Authors:** B. I. B. Lindahl

**Affiliations:** Department of Philosophy, Stockholm University, 106 91 Stockholm, Sweden

**Keywords:** Causal selection, COVID-19, Prevention, Underlying cause of death

## Abstract

The World Health Organization has issued international instructions for certification and classification (coding) of the coronavirus disease (COVID-19) as cause of death. Central to these instructions is the selection of the underlying cause of death for a public health preventive purpose. This article focuses on two rules for this selection: (1) that a death due to COVID-19 should be counted independently of pre-existing conditions that are suspected of triggering a severe course of COVID-19 and (2) that COVID-19 should not be considered as due to anything else. The article argues that observance of the first rule may not always lead to an optimal selection from a preventive point of view and that in the future the ascertainment of an animal source of the coronavirus (SARS-CoV-2) would make it possible to reconceptualize ‘COVID-19′ and create a zoonotic classification code by means of which a factor of a greater preventive value could be selected than what is currently possible.

In this journal Maria Cristina Amoretti and Elisabetta Lalumera (2021) addressed the question: When does COVID-19 qualify as *the underlying cause of death*? They called attention to the preventive public health purpose of national cause-of-death statistics — emphasized by the World Health Organization (WHO) — and to WHO’s rigorous instructions for physicians on cause-of-death certification and for the subsequent statistical classification (coding) of these data. Amoretti and Lalumera highlighted several uncertainties regarding these instructions. In the present article I will focus on two issues in their paper: first, the problem of selecting the underlying cause of death in cases of comorbidity, where, according to WHO, a death due to COVID-19 “should be counted independently of preexisting conditions that are suspected of triggering a severe course of COVID-19” (WHO 2020, p. 3) and second, the problem of deciding whether a causal relationship is acceptable or not for mortality coding — a decision “founded not only on a medical assessment but also on epidemiological and public health considerations” (WHO 2018, 2.18.2).^1^

Studies of people with COVID-19 have shown that cancer, cardiovascular disease, chronic respiratory disease, diabetes, hypertension and older age were all associated with an increased risk of death (Jordan et al. 2020). This was not unexpected perhaps, since these conditions are all known to be significant risk factors even without COVID-19. But it makes it reasonable from a preventive point of view to question WHO’s instruction that in the selection of the underlying cause of death a death due to COVID-19 should be counted independently of pre-existing conditions that are suspected of triggering a severe course of COVID-19. Cancer is an illustrative example.

Although conflicting results have been reported on the risk of death in COVID-19 patients with cancer (Albiges et al. 2020), in some cases it seems as if the course of the COVID-19 and that of the cancer have consequences that reciprocally interact, increasing the seriousness of the outcome: severe COVID-19 “is linked to an inflammatory burst and lymphopenia, which may aggravate cancer prognosis” and cancer predisposes to severe COVID-19 because “antineoplastic therapies such as surgery, chemotherapy and radiotherapy may debilitate the immune system and cause immunosenescence and inflammaging” (Derosa et al. 2020, pp. 946, 959).

Clearly, in cases where there are such interactions, the WHO’s instruction that a death due to COVID-19 should be counted independently of pre-existing conditions need some further justification, in addition to the stated purpose of prevention. On first inspection, in such cases it does not seem unreasonable to claim that the cancer and the COVID-19 *jointly* initiated the train of morbid events leading directly to death. But the WHO’s instructions do not allow of this solution. Another option would be to apply a broader interpretation of “the train of morbid events” and argue that the *cancer* initiated a relevant train of events before the COVID-19 appeared. One may then argue also that prevention of cancer would reduce the risk of death in people with COVID-19. In both alternatives the selection of the cancer as the underlying cause of death would serve the purpose of prevention. But, of course, prevention of cancers may require a much longer public health time perspective than prevention of infections. It is important to keep in mind, however, that underlying cause of death statistical classification (coding) (e.g., of cancer) for a long-term preventive purpose need not rule out implementation of short-term preventive measures (e.g., regarding COVID-19).

When it comes to the problem of deciding whether a causal relationship is acceptable or not for mortality coding, WHO’s instructions provide that “COVID-19 is not considered as due to, or as an obvious consequence of, anything else” and this “in analogy to the coding rules applied for INFLUENZA” (WHO 2020, p. 8). The WHO does not exclude antecedents of infectious diseases of *all* kinds, however. A cause of an HIV infection — a preceding condition necessitating blood transfusion (e.g., haemophilia, anaemia and major injury), an invasive procedure (e.g., surgery) and drug abuse — should be accepted when selecting the underlying cause of death (WHO 2016b, p. 56, 2018, 2.21.1.2). The difference might simply be a matter of taking into account only sufficiently well known risk factors that fit into the WHO classification (ICD).

It is of interest to note here that for a certain type of hypersensitivity pneumonitis — the disease *bird fancier’s lung* — the ICD-10 and ICD-11 provide a *zoonotic* code, partly referring to allergens from birds (WHO 2016a, 2018). In a similar way, given that an animal source of the coronavirus (SARS-CoV-2) will be sufficiently established, a zoonotic ICD code could be created that would include a reference to the animal source by a reconceptualization of ‘COVID-19′.^2^ By means of such a code a factor of a greater public health preventive value could be selected as the underlying cause of death than what the present, provisional, codes for COVID-19 — U07.1, virus identified and U07.2, virus not identified (WHO 2020) — make possible. By indicating the source of the virus, a zoonotic COVID-19 code would make possible early preventive measures against the source itself.

The problem of selecting, in an individual case, a single cause from among several established causes as the principal cause of a certain effect, is a problem that has been discussed at least since John Stuart Mill’s thorough analysis in *A System of Logic* (1843). The problem of causal attribution appears not only in the contexts of medicine and cause-of-death statistics, but also in other professional fields, such as history, law and natural science, as well as in everyday life (for a review, see Lindahl 2009). Different aspects of researcher positionality (Holmes 2020) may influence the making of causal attributions. From this point of view, it may be argued that even the suggestions for conceptual and causal theoretical improvements made in the present article are influenced by such factors as the author’s particular philosophical and public health research background. It should be noted, however, that my suggestions are in line with WHO’s overall purpose of prevention.

The attempts to formulate international cause-of-death selection rules for statistical purposes may be traced back to the beginning of the twentieth century (Lindahl 1988). Over the years the selection principles have become increasingly complex and in order to ensure a consistent procedure several countries implement computer software for the selection of the underlying cause of death (Johansson 2008). The efforts to further improve the selection procedure will certainly continue. As we have seen, the WHO’s rules may not always yield an optimal result from a preventive point of view. In cases of partly parallel reciprocally interacting sequences — one with COVID-19, another with cancer — selecting the cancer could be more effective. And, possibly, for COVID-19 sequences, the selection could be improved by creating a zoonotic ICD code, covering both COVID-19 and a source of the virus.

In a wider perspective there are a number of more general difficulties in public health preventive work (Allander and Lindahl 1997), some of which are relevant to causal attribution of deaths in people with COVID-19. One should keep in mind that cause-of-death statistics and the register on which they are based, are sources for aetiological research — the results of which may identify an optimal point for effective public health intervention.

## Footnotes

1 Regarding the purpose and definition of the notion of the underlying cause of death, WHO states: “Effective public health interventions prevent harm or death by breaking the chain of events that lead to harm. For this purpose, the underlying cause of death has been defined as ‘(a) the disease or injury which initiated the train of morbid events leading directly to death, or (b) the circumstances of the accident or violence which produced the fatal injury’, and is selected for routine single-cause tabulation of mortality statistics.” (WHO 2018, 2.17.1).

2 Based on genetic comparisons of viruses detected in bats and pangolins, various scenarios for the origin of the coronavirus and for the zoonotic transmission route of this and related viruses have been suggested (Banerjee et al. 2021).
